# Clinical trials of new drugs for Alzheimer disease: a 2020–2023 update

**DOI:** 10.1186/s12929-023-00976-6

**Published:** 2023-10-02

**Authors:** Li-Kai Huang, Yi-Chun Kuan, Ho-Wei Lin, Chaur-Jong Hu

**Affiliations:** 1 PhD Program in Medical Neuroscience, College of Medical Science and Technology, Taipei Medical University, No. 291, Zhong Zheng Road, Zhonghe District, New Taipei City, Taiwan; 2 Taipei Neuroscience Institute, Taipei Medical University, New Taipei City, Taiwan; 3 Dementia Center and Department of Neurology, Shuang-Ho Hospital, Taipei Medical University, New Taipei City, Taiwan; 4 Department of Neurology, School of Medicine, College of Medicine, Taipei Medical University, Taipei, Taiwan; 5 Department of Biomedical Engineering, National Taiwan University, Taipei, Taiwan; 6 School of Medicine, College of Medicine, Taipei Medical University, Taipei, Taiwan

**Keywords:** Alzheimer disease, Clinical trials, Anti-amyloid, Anti-tau, Neuroprotection, Cognitive enhancement, Neuroinflammation

## Abstract

Alzheimer's disease (AD) is the leading cause of dementia, presenting a significant unmet medical need worldwide. The pathogenesis of AD involves various pathophysiological events, including the accumulation of amyloid and tau, neuro-inflammation, and neuronal injury. Clinical trials focusing on new drugs for AD were documented in 2020, but subsequent developments have emerged since then. Notably, the US-FDA has approved Aducanumab and Lecanemab, both antibodies targeting amyloid, marking the end of a nearly two-decade period without new AD drugs. In this comprehensive report, we review all trials listed in clinicaltrials.gov, elucidating their underlying mechanisms and study designs. Ongoing clinical trials are investigating numerous promising new drugs for AD. The main trends in these trials involve pathophysiology-based, disease-modifying therapies and the recruitment of participants in earlier stages of the disease. These trends underscore the significance of conducting fundamental research on pathophysiology, prevention, and intervention prior to the occurrence of brain damage caused by AD.

## Background

Alzheimer disease (AD) represents a major global medical, social, and economic burden. The World Alzheimer Report 2022 revealed that more than 55 million people have AD or related conditions worldwide, and this number is projected to reach 82 million by 2030 and 138 million by 2050 [1]. Typically, AD first manifests as progressive memory decline accompanied or followed by other cognitive dysfunctions, such as visuospatial abnormalities, navigation difficulties, executive problems, and language disturbances. These cognitive impairments affect the performance of activities of daily living. During the course of AD, many behavioral and psychological symptoms of dementia (BPSD) occur [2–4].

Although the exact causes of AD remain unclear, the disease has two pathological hallmarks: plaques composed of amyloid-beta (Aβ) fibrils and neurofibrillary tangles (NFTs) consisting of hyperphosphorylated tau protein [5–7]. The key event in AD pathogenesis is believed to be Aβ accumulation. Cerebral Aβ fibril deposition may occur decades before the onset of clinical symptoms [8]. Brain atrophy, particularly in the hippocampus, is major indicator of early Aβ accumulation, particularly in the presubiculum [9, 10]. Aβ accumulation was discovered to be crucial by three independent research groups in 1991 [11–13]. In familial AD, mutant autosomal-dominant genes, including the genes for amyloid precursor protein (*APP*), presenilin-1 (*PSEN1*), and presenilin-2 (*PSEN2*), encode the major proteins involved in amyloid metabolism [13–15]. Individuals with trisomy 21 (Down syndrome) have an extra copy of the *APP* gene, which may result in increased amyloid production and AD risk in middle age [16]. At present, the predominant theory regarding the cause of AD is the amyloid hypothesis; crucial advancements in AD therapy have been made on the basis of the proposed role of amyloid accumulation in the AD development. The United States Food and Drug Administration (US FDA) granted traditional approval for Leqembi (lecanemab-irmb) on July 6, 2023, for the treatment of AD [17]. The approval of this treatment not only affirms the pathophysiological significance of amyloid in AD but also marks a notable advance in clinical approaches to AD treatment, remedying the scarcity of new drugs in the market for nearly two decades.

Tau is a microtubule-associated protein that aids in microtubule assembly and stabilization. In AD, tau becomes hyperphosphorylated and aggregates to form paired helical filaments, a major component of NFTs within the neuronal cytoplasm. As the disease progresses, the gradual spread of tau pathology throughout brain regions has been suggested to be caused by the transfer of abnormal types of tau protein from one neuron to another [18]. The accumulation of NFTs might be initiated between the accumulation of Aβ and the development of clinical symptoms of AD [19]. NFTs and quantitative neuronal loss may be more closely correlated with disease severity and dementia progression than the amyloid plaque burden [20–22]. Positron emission tomography (PET) investigations have revealed a strong correlation between the binding characteristics of tau tracers and the severity of clinical manifestations in individuals with AD [23]. Molecular imaging modalities (PET) and cerebrospinal fluid (CSF) and blood–based biomarkers have extended the diagnostic scope of AD pathology to both clinical and even preclinical settings. The analysis of a combination of biomarkers such as amyloid, tau, and neurodegeneration (collectively, ATN classification) has been proposed by research on AD [24, 25]. Furthermore, the exceptional diagnostic accuracy of plasma-based biomarkers has facilitated the clinical transition of fluid biomarkers from research settings to clinical practice. A recent presentation at the Alzheimer’s Association International Conference in 2023 highlighted the clinical and research applications of two fundamental AD biomarker categories, labeled as A and T. The A category pertains to biomarkers associated with the Aβ proteinopathy pathway, and the T category pertains to biomarkers linked to tau proteinopathy [26].

Aβ serves as a proinflammatory agent and triggers the nuclear factor κB (NF-κB) pathway in astrocytes, increasing complement C3 release. Subsequently, by binding to C3a receptors, C3 causes neuronal dysfunction and microglial activation [27]. In the early stage of AD, activated microglia may play a protective, anti-neuroinflammatory role by clearing amyloids and releasing nerve growth factors. However, activated microglia induce neurotoxic A1 astrocyte reactivity through the release of IL-1α, C1q, and TNF-α, resulting in a feedback loop of dysregulated inflammation in AD [28]. The excessive accumulation of Aβ or other toxic compounds activates proinflammatory phenotypes, resulting in neuronal damage [29]. Sustained inflammation has been observed in the brains of patients with AD [30, 31]. The inadequate clearance of Aβ along with the aggregation of tau disrupts microglial defense mechanisms, resulting in sustained and harmful microglial activation [32]. The sequential occurrence of amyloid plaque formation, microglial activation, and the pathological phosphorylation and aggregation of tau proteins to form NFTs is the fundamental notion of the amyloid cascade–inflammation hypothesis. In the Multi-Ethnic Study of Atherosclerosis (multiple covariates were controlled for), vascular risk factor profiles and Aβ deposition significantly predicted cognitive decline [33]. Vascular risk factors can also lead to inflammation in the brain, which damages neuronal cells and further increases the likelihood of AD dementia [34].

The role of autophagy impairment is proposed in a novel hypothesis concerning plaque formation in AD. Among neurons that are compromised but still maintain some integrity, autophagic vacuoles (AVs) containing abundant Aβ are notably present. These AVs cluster within expansive membrane blebs, exhibiting a distinctive flower-like arrangement termed PANTHOS. These formations constitute the primary source of the majority of amyloid plaques found in mouse models of AD [35]. Neuroprotective therapies, including free radical scavengers, regeneration enhancers, and the suppression of excitable amino acid signaling pathways, have been proposed for preventing neuronal death or brain atrophy caused by amyloid, tau, and neuroinflammation [36]. Pathological evidence indicates that AD is also associated with degeneration in cholinergic neuron-rich regions, such as the nucleus basalis of Meynert, frontal cortex, and anterior and posterior cingulate cortex, which can lead to the symptoms of memory impairment and agitation. Acetylcholine (ACh) plays a vital role in memory function, including memory encoding, consolidation, and retrieval processes, and increasing Ach levels by using cholinesterase inhibitors (AChEIs) has become a standard therapy for the symptoms of AD [37].

**Fig. 1:**
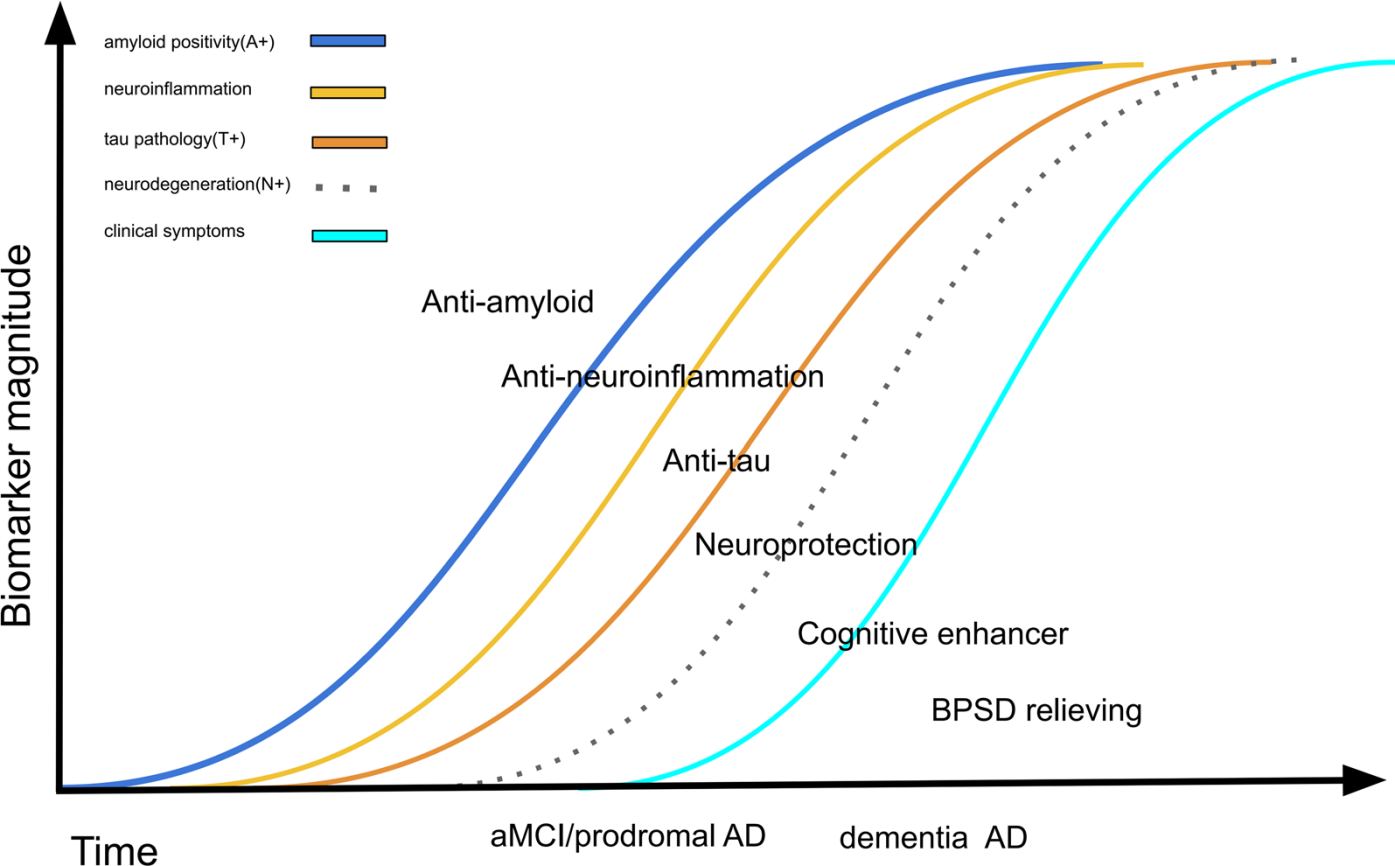
According to the amyloid hypothesis, the pathophysiology and clinical course of Alzheimer's disease progress as follows: amyloid accumulation, neuroinflammation, tau accumulation, brain metabolism dysfunction, brain atrophy, cognitive decline (from mild cognitive impairment to dementia), and the development of dementia symptoms. Novel drugs should target at least one of these events. *AD* Alzheimer's disease, *aMCI* amnestic mild cognitive impairment, *BPSD* behavioral psychological symptoms of dementia

Clinical trials of early or preventive interventions based on amyloid/tau theory and those targeting other pathophysiologies are ongoing or have been initiated. Many ongoing clinical trials on AD are focused on disease-modifying therapies (DMTs) that target the causes and can change the course of AD. The other trials involve symptomatic treatments—for example, enhancing cognitive function and relieving BPSD (Fig. 1). In this review, we summarize the new drugs being examined in ongoing trials (listed on ClinicalTrials.gov) and discuss the trends in and obstacles in AD clinical trials.

### Anti-amyloid therapy

Table 1 summarizes the US FDA approval status of anti-amyloid agents. Tables 2 and 3 summarize the ongoing phase 3 and phase 2 trials of anti-amyloid therapy respectively.

**Table 1:** Anti-amyloid therapy US FDA–approval status

Name	Synonyms	Company	Target	Therapy type	Approval status
Aduhelm	Aducanumab, BIIB037	Biogen, Neurimmune	Aβ aggregates	Immunotherapy (passive)	Accelerated approval
Leqembi	Lecanemab-irmb, BAN2401	BioArctic AB, Biogen, Eisai	Aβ protofibrils	Immunotherapy (passive)	Full approval
Donanemab	N3pG-Aβ Monoclonal Antibody, LY3002813	Eli Lilly & Co	Aβ aggregates	Immunotherapy (passive)	Application for full approval

**Table 2:** Ongoing phase 3 trials of anti-amyloid therapy

Agent	Company	Synonyms	Therapeutic purpose	ClinicalTrials.gov identifier	Status
Aducanumab (EMBARK)	Biogen, Neurimmune	BIIB037	Immunotherapy (passive)	NCT04241068	Active, not recruiting
Aducanumab (ENVISION)			Immunotherapy (passive)	NCT05310071	Recruiting
Donanemab (TRAILBLAZER-ALZ 2)	Eli Lilly & Co	N3pG-Aβ Monoclonal Antibody	Immunotherapy (passive)	NCT04437511	Active, not recruiting
Donanemab (TRAILBLAZER-ALZ 3)			Immunotherapy (passive)	NCT05026866	Recruiting
Donanemab|/Aducanumab (TRAILBLAZER-ALZ 4)			Immunotherapy (passive)	NCT05108922	Active, not recruiting
Donanemab (TRAILBLAZER-ALZ 5)			Immunotherapy (passive)	NCT05508789	Recruiting
Donanemab (TRAILBLAZER-ALZ 6)			Immunotherapy (passive)	NCT05738486	Recruiting
Lecanemab (Clarity AD)	BioArctic AB, Biogen, Eisai	Lecanemab-irmb	Immunotherapy (passive)	NCT03887455	Active, not recruiting
Lecanemab (AHEAD 3–45 Study)			Immunotherapy (passive)	NCT04468659	Recruiting
Lecanemab and E2814 DIAN-TU-001 (E2814)	Eisai		Immunotherapy (passive)	NCT05269394	Recruiting
Remternetug (TRAILRUNNER-ALZ1)	Eli Lilly & Co	LY3372993	Immunotherapy (passive)	NCT05463731	Recruiting
Solanezumab (A4)	Eli Lilly & Co	LY2062430	Immunotherapy (passive)	NCT02008357	Active, not recruiting
Solanezumab/ gantenerumab (DIAN-TU-001)			Immunotherapy (passive)	NCT01760005	Recruiting
ALZ-801 (APOLLOE4)	Alzheon	valiltramiprosate	Small molecule	NCT04770220	Active, not recruiting
Simufilam (RETHINK-ALZ)	Cassava Sciences	PTI-125	Small molecule	NCT04994483	Recruiting
Simufilam (REFOCUS-ALZ)			Small molecule	NCT05026177	Recruiting
Simufilam			Small molecule	NCT05575076	Enrolling through invitation

**Table 3:** Ongoing phase 2 anti-amyloid therapy trials

Agent	Company	Synonyms	Therapeutic purpose	ClinicalTrials.gov identifier	Status
ABBV-916	AbbVie	N3pG-Abeta mAb	Immunotherapy (passive)	NCT05291234	Recruiting
ALZ-801	Alzheon	valiltramiprosate	Small molecule	NCT04693520	Active, not recruiting
CT1812 (SHINE (COG0201) Study)	Cognition Therapeutics	Elayta	Small molecule	NCT03507790	Recruiting
CT1812 (START(COG0203) study)				NCT05531656	Recruiting
Varoglutamstat (VIVA-MIND)	Vivoryon Therapeutics AG. ADCS. NIA	PQ912	Small molecule	NCT03919162	Recruiting
Varoglutamstat (VIVID)				NCT04498650	Active, not recruiting

Aducanumab (brand name: Aduhelm) is a high-affinity, fully human immunoglobulin gamma 1 (IgG1) monoclonal antibody that binds to the N-terminus of Aβ fibrils and blocks amyloid aggregation [38]. In August 2015, two phase 3 clinical trials, namely ENGAGE and EMERGE studies, were initiated. These trials compared monthly intravenous infusions of aducanumab at one of three doses with infusions of placebo over 18 months, and the primary outcomes were cognitive and functional decline, which were assessed using the Clinical Dementia Rating (CDR) scale Sum of Boxes (CDR-SB). The secondary outcomes were other cognitive and functional measures. The trials were conducted in 150 centers across North America, Europe, Australia, and Asia. However, the findings of the EMERGE trial reached statistical significance, whereas the primary endpoint was not reached in the ENGAGE trial. An exploratory analysis revealed that a subgroup of the participants in the ENGAGE trial who received a high dose of aducanumab exhibited slow decline, which was similar to that observed among the participants in the EMERGE trial. The US FDA approved aducanumab in June 2021 on the basis of the data of the EMERGE and ENGAGE trials. Both trials presented evidence of an intermediate effect of the drug on biomarkers, indicating amyloid removal, which is likely linked to the clinical benefit of aducanumab. Further trials must be conducted to confirm the potential benefit of aducanumab [39]. The phase 3b/4 ENVISION trial (NCT05310071), which began in 2022, will enroll 1,512 patients with early AD who will receive either monthly doses of aducanumab of up to 10 mg/kg or placebo for 18 months. The aim of the trial is to determine the efficacy of aducanumab in delaying cognitive and functional decline in comparison with placebo, which would be determined on the basis of CDR-SB scores. The secondary endpoints of the trial include scores on the Alzheimer’s Disease Assessment Scale–Cognitive Subscale (ADAS-Cog) 13, Alzheimer’s Disease Cooperative Study–Activities of Daily Living Inventory (ADCS-ADL)–Mild Cognitive Impairment Version, Integrated Alzheimer’s Disease Rating Scale (iADRS), Mini-Mental State Examination, and Neuropsychiatric Inventory. The trial intends to recruit 18% of its participants from Black and Latinx populations in the United States and will have a long-term follow-up of up to 4 years, with results expected by 2026. The EMBARK trial (NCT04241068) is a phase 3b open-label study including 1,696 participants from previous aducanumab trials (from trials 221AD103, 221AD301, 221AD302, and 221AD205) that will assess aducanumab safety and tolerability over 100 weeks after a wash-out period. Participants will receive an intravenous infusion of aducanumab at 10 mg/kg monthly for 2 years, and eligible participants will continue to receive the infusion for another 52 weeks during the long-term extended treatment period. The primary outcomes are safety and tolerability, and the efficacy endpoints are the same as those in the EMERGE and ENGAGE trials, and Caregiver Global Impression of Change evaluations will be conducted every 6 months. All participants will undergo volumetric magnetic resonance imaging (MRI) scans, and a subset of the study population will undergo biomarker testing, including amyloid PET, tau PET, and CSF testing.

Lecanemab (brand name: Leqembi), a humanized IgG1 antibody derived from mAb158, selectively binds to soluble Aβ protofibrils [40]. The US FDA approved it on January 6, 2023, through an accelerated approval pathway on the basis of evidence of amyloid removal in a phase 2 trial (NCT01767311) and because it had a likelihood of having clinical benefits [41] A double-blind, placebo-controlled phase 2 trial recruited 856 patients with AD with mild cognitive impairment (MCI) or mild dementia and verified amyloid pathology through amyloid PET or CSF Aβ1-42 [42]. The results revealed a significant and dose-dependent reduction of amyloid plaques in the lecanemab group (10 mg/kg, intravenous infusion every 2 weeks) from baseline to week 79 compared with the placebo group. At the time of writing this paper, three phase 3 clinical trials on lecanemab are underway. The first trial, Clarity AD (NCT03887455), was initiated in March 2019 and was conducted at 250 sites around the world. It reported favorable outcomes for all primary and secondary measures, including ADAS-Cog14, AD Composite Score (ADCOMS), and ADCS-MCI-ADL scores [43]. The second trial is AHEAD 3–45 (NCT04468659), which was initiated in July 2020 as a 4-year trial comprising two substudies, one of which is A3, and the other one is A45. A3 is enrolling 400 people whose amyloid levels are below the brain-wide threshold for positivity; participants will receive 5 mg/kg lecanemab titrated to 10 mg/kg or placebo every month for 216 weeks. A45 is enrolling 1,000 cognitively healthy participants with positive amyloid PET scans, and they will receive lecanemab titrated to 10 mg/kg every 2 weeks for 96 weeks, followed by 10 mg/kg every month through week 216. The trial is expected to run until October 2027. The third phase 3 clinical trial is the Dominantly Inherited Alzheimer Network Trials Unit (DIAN-TU) Next Generation trial (DIAN-TU-001 (E2814), NCT05269394), in which a combination of lecanemab and the anti-tau antibody E2814 (phase 2) will be administered to 168 people with familial AD mutations. On July 6, 2023, Leqembi (lecanemab-irmb) received traditional approval from the US FDA for the treatment of AD based on Phase 3 data from the Clarity AD clinical trial [17].

The appropriate use recommendations (AURs) for lecanemab and aducanumab highlight the importance of patient selection, surveillance for adverse events, and clinician preparedness [44, 45]. The AURs for both drugs have several similarities with respect to age criteria, biomarker requirements (positive amyloid PET or CSF findings indicative of AD), diagnosis (MCI due to AD or mild AD dementia), and MRI exclusion criteria (e.g., microhemorrhages and cortical infarction). The AURs also emphasize the importance of monitoring for amyloid-related imaging abnormalities (ARIAs), which can occur in patients receiving these drugs. *APOE* genotyping is recommended for informing risk discussions with candidate participants because *APOE4* allele carriers, especially *APOE4* homozygotes, are at a high risk of ARIAs. Patients receiving treatment must have care partners or family members who can provide necessary support and who clearly understand the nature and requirements of the therapy. Discontinuation of treatment is recommended in the following situations: when a patient is taking drugs with associated risks, such as anticoagulation agents for conditions like atrial fibrillation, deep vein thrombosis, or pulmonary embolism; or when any of the following conditions occur: a hypercoagulable state, or the development of any of the following: cerebral macrohemorrhage, multiple areas of superficial siderosis, more than 10 instances of microhemorrhages since treatment initiation, severe symptoms of ARIAs, or two or more episodes of ARIAs.

Donanemab is a humanized monoclonal antibody developed from mouse mE8-IgG2a. It recognizes Aβ (3–42), an aggregated form of Aβ found in amyloid plaques [46]. It was discovered to be bound to approximately one-third of amyloid plaques in postmortem brain samples of patients with AD or Down syndrome, and it strongly reacted with the plaque core [47]. In the phase 2 TRAILBLAZER-ALZ study, the safety, tolerability, and efficacy of donanemab alone and in combination with the Beta-Secretase 1 (BACE1) inhibitor LY3202626 (developed by Eli Lilly and Company) were evaluated over 18 months. The trial met its primary endpoint of delaying decline—which was determined on the basis of iADRS scores—by 32% compared with placebo. Amyloid burden reduction was correlated with improvement in iADRS scores only in *ApoE4* carriers [48]. Donanemab reduced the tau burden in the temporal, parietal, and frontal lobes and significantly decreased plasma pTau217 by 24% in the treatment group, whereas the placebo group exhibited a 6% increase in plasma pTau217 at the end of the trial [49]. At the time of writing this paper, five phase 3 trials of donanemab are underway: TRAILBLAZER-ALZ 2, TRAILBLAZER-ALZ 3, TRAILBLAZER-ALZ 4, TRAILBLAZER-ALZ 5 and TRAILBLAZER-ALZ 6. The TRAILBLAZER-ALZ 2 (NCT04437511) trial was initially started in June 2020 as a phase 2 safety and efficacy trial, and 500 patients with early AD were recruited. Inclusion criteria of TRAILBLAZER-ALZ 2 are similar to those of TRAILBLAZER-ALZ: a ≥ 6-month history of worsening memory and positive amyloid (flortaucipir) PET. The trial was subsequently extended to a phase 3 trial with 1,800 participants. The primary outcome is iADRS, and the effectiveness of treatment is being measured using a disease-progression model rather than solely on the basis of changes at the final time point. Trial results for 1,736 participants were published to report donanemab’s impact on early symptomatic AD. Using PET imaging to categorize individuals into groups with low/medium or high tau pathology load, the study spanned 18 months and assessed cognitive and functional scales. Donanemab achieved significant cognitive improvement in the low/medium tau group (iADRS change: − 6.02 vs. − 9.27 placebo) and combined population (change: − 10.2 vs. − 13.1 placebo). The drug notably reduced decline by 60% in patients with early-stage AD, supporting the efficacy of short-term dosing. Twenty-four outcomes were evaluated, with significant findings for 23 outcomes. Adverse effects included amyloid-related imaging problems (24% donanemab vs. 2.1% placebo) and infusion-related reactions (8.7% donanemab vs. 0.5% placebo). The study findings indicated the potential of donanemab to slow AD progression, particularly in the early stage [50]. In the TRAILBLAZER-ALZ study, donanemab slowed disease progression by 32% at 18 months (*p* = 0.04 vs. placebo), thus demonstrating clinical efficacy [51]. TRAILBLAZER-ALZ 3 (NCT05026866) is a placebo-controlled phase 3 prevention trial that was started in August 2021. The trial plans to enroll 3,300 cognitively healthy people aged 50–55 years who are at high risk of AD, as determined by elevated plasma pTau217 levels and Telephone Interview for Cognitive Status-modified scores. The primary outcome is the time to clinical progression, which is measured using global CDR scores. Participants are to be monitored every 6 months until cognitive impairment is noted (i.e., a score above 0 on the CDR for two consecutive evaluations) in 434 participants. The trial has a decentralized design and is being conducted at more than 200 sites in the United States, Japan, and Puerto Rico until November 2027. TRAILBLAZER-ALZ 4 (NCT05108922) is a phase 3, open-label, head-to-head comparison of amyloid clearance by either donanemab or aducanumab that began in November 2021 after the US FDA approval of aducanumab. The trial enrolled 200 people with early symptomatic AD, as indicated by a global CDR score of 0.5 or 1, at 31 sites in the United States. The primary outcome is the percentage of participants who achieve complete amyloid plaque clearance after 6 months for each treatment group, with clearance determined using amyloid (florbetapir) PET. The trial has 17 secondary outcomes, which are all related to amyloid PET measurements at up to 18 months. The preliminary results were presented at the 2022 Clinical Trial of AD (CTAD) conference: 38% of the patients on donanemab exhibited amyloid levels below the amyloid positivity threshold after 6 months, whereas only 2% of the patients on aducanumab has such findings. Plasma pTau217 levels decreased by 25% for the participants receiving donanemab, but not at all for those receiving aducanumab. The side effect of ARIA-edema occurred in 22% of the participants in both groups. TRAILBLAZER-ALZ 5 (NCT05508789) is being conducted to assess the safety and efficacy of donanemab in individuals with early symptomatic AD. The trial started in October 2022; 1,500 participants will be recruited by using the same criteria as those of TRAILBLAZER-ALZ 2 from 148 sites across China, Korea, Taiwan, and Europe; and the trial is expected to run until mid-2025. Participants will be administered monthly infusions of either donanemab or placebo, and the primary outcome will be measured on the basis of iADRS score changes after 18 months. TRAILBLAZER-ALZ 6 (NCT05738486) is a phase 3b study that will assess the impact of various dosing regimens of donanemab on the occurrence and severity of ARIA-E (ARIA with edema or effusion) in 800 adults with early symptomatic AD. The study also seeks to identify participant characteristics that predict the risk of ARIA-E. The trial is divided into four arms, each with a distinct donanemab dose.

Remternetug is a monoclonal antibody that recognizes a pyroglutamated form of Aβ that aggregates into amyloid plaques. In August 2022, Eli Lilly and Company initiated a phase 3 trial called TRAILRUNNER-ALZ1 (NCT05463731) that will randomize 600 patients with early symptomatic AD across 75 sites in the United States and 2 sites in Japan into groups receiving the antibody or placebo through intravenous infusion or subcutaneous injection for 1 year. The primary outcome is the percentage of patients whose amyloid plaques are cleared by the end of the treatment period. The secondary outcomes include the measurement of amyloid clearance, pharmacokinetics, and treatment-emergent anti-drug antibodies. The study also plans to conduct a year-long, blinded crossover extension. An additional safety cohort of 640 patients will receive open-label remternetug for 1 year.

Solanezumab is a humanized monoclonal antibody that targets the mid-domain of the Aβ peptide for increasing Aβ clearance [52]. Phase 3 trials of solanezumab, including EXPEDITION-1 and EXPEDITION-2, which enrolled 2,052 patients with mild-to-moderate AD, did not reveal improvements in ADAS-Cog11 and ADCS-ADL scores, which were the primary outcome measures. Similarly, the phase 3 trial EXPEDITION-3 demonstrated that 400 mg solanezumab administered every 4 weeks did not have significant effects on cognitive decline in patients with mild AD [52]. A4 (NCT02008357) is a phase 3 prevention trial focused on slowing memory and cognitive decline in elderly individuals without cognitive impairment or dementia. A4 is using a sensitive cognitive battery—the Alzheimer Disease Cooperative Study Preclinical Alzheimer Cognitive Composite—and was initiated in February 28, 2014. On March 8, 2023, Eli Lilly and Company reported that solanezumab did not slow cognitive decline or clear amyloid plaques in individuals with preclinical AD in the A4 study. DIAN-TU-001 (NCT01760005) is another ongoing phase 3 clinical trial that is testing the combination of solanezumab and gantenerumab in 210 asymptomatic and mildly symptomatic carriers of autosomal-dominant mutations in AD genes. However, on February 10, 2020, the study investigators announced that the primary endpoint was not achieved in the trial, namely treatment-related changes on the DIAN-Multivariate Cognitive Endpoint. The results indicated that the solanezumab-treated group had greater cognitive decline on some measures relative to placebo, and that solanezumab treatment did not exert any beneficial effects on downstream biomarkers, whereas gantenerumab significantly reduced amyloid plaques, CSF total tau, and phospho-tau181 and attenuated increases in neurofilament light chain [53]. The participants were offered an open-label extension with high-dose gantenerumab because of its positive effects on imaging and other biomarkers, such as normalized CSF Aβ42, and because it reduced CSF total tau and pTau181 levels.

ALZ-801 is a prodrug of tramiprosate, a small molecule of anti-Aβ oligomers and an aggregation inhibitor [54]. The phase 3 trial APOLLOE4 (NCT04770220) is evaluating the safety and efficacy of ALZ-801 for patients with early AD and carrying the homozygous ε4 allele on the apolipoprotein E gene (*APOE4/4*). The recruited patients are receiving 265 mg ALZ-801 or placebo twice daily for 18 months. The trial started in May 2021. The primary endpoint is ADAS-Cog scores, and the secondary endpoints are scores of the Disability Assessment for Dementia, CDR-SB, and Amsterdam-iADL. The biomarkers of interest include the hippocampal volume, as determined through MRI and based on CSF and plasma pTau181 levels. Another phase 2 trial (NCT04693520) is investigating the effects of oral ALZ-801 administered to participants with early AD who have the *APOE4/4* or *APOE3/4* genotype with biomarkers of core AD pathology. The study is also assessing the efficacy, safety, and tolerability of ALZ-801.

Simufilam (PTI-125) is a drug that binds to filamin, a scaffolding protein that stabilizes the interaction between soluble Aβ42 and the α7 nicotinic acetylcholine receptor [55]. Two phase 3 trials, namely RETHINK-ALZ (NCT04994483) and REFOCUS-ALZ (NCT05026177), were commenced in November 2021. Both are safety and efficacy studies of simufilam and have enrolled participants with mild-to-moderate AD. RETHINK-ALZ will randomize 750 participants with AD and CDR scores of 0.5, 1, or 2 into groups receiving either placebo or 100 mg of simufilam twice a day for 1 year (52 weeks). The coprimary outcomes of this trial are ADAS-Cog12 and ADCS-ADL scores, and the trial is set to run through October 2023. REFOCUS-ALZ will randomize 1,083 participants into groups receiving placebo or 50 or 100 mg of simufilam (1:1:1) for 76 weeks. The primary outcome measures are similar to those of the RETHINK-ALZ trial. A phase 3 trial of simufilam (NCT05575076) was started in November 2022 to assess the long-term safety and tolerability of simufilam in participants with mild-to-moderate AD. That open-label extension study is intended to assess the long-term safety and tolerability of simufilam 100 mg twice daily in patients who have completed the RETHINK-ALZ or REFOCUS-ALZ Phase 3 clinical trials. The primary outcome measure is adverse event monitoring from baseline to week 52.

Varoglutamstat (PQ912) is a glutaminyl cyclase inhibitor that reduces pGlu-Aβ generation [56]. Glutaminyl cyclase catalyzes the cyclization of an exposed glutamate at the N-terminus of Aβ, resulting in the formation of toxic pGlu-Aβ, a major component of amyloid plaques. Two ongoing phase 2 clinical trials, namely VIVA-MIND and VIVIAD, are evaluating the safety, tolerability, and efficacy of varoglutamstat in participants with MCI and mild dementia due to AD. VIVA-MIND (NCT03919162) is a phase 2A multicenter, randomized, double-blind, placebo-controlled, parallel-group study of varoglutamstat, with a stage gate to phase 2B. Phase 2A involves an adaptive dosing evaluation of three doses of varoglutamstat or placebo for ≥ 24 weeks. VIVIAD (NCT04498650) is a phase 2B, multicenter, randomized, double-blind, placebo-controlled, parallel-group, dose-finding study being conducted to evaluate the safety, tolerability, and efficacy of varoglutamstat in 259 participants with MCI and mild dementia due to AD.

ABBV-916 is a monoclonal antibody to Aβ. It recognizes N-terminal truncated Aβ modified with pyroglutamate at position 3 (N3), a form of Aβ that is aggregated into amyloid plaques. A two-stage phase 2 trial of ABBV-916 is ongoing (NCT05291234). Stage A is a multiple ascending dose study, and participants have a 25% chance of receiving placebo. Stage B is a proof-of-concept study, and participants have a 20% chance of receiving placebo. The first 6 months of the study are a double-blinded period, which is to be followed by a 2-year extension period in which all participants receive ABBV-916. Approximately 195 participants aged 50–90 years are to be enrolled at approximately 90 sites across the world. The participants are to receive intravenous doses of ABBV-916 or placebo once every 4 weeks for 24 weeks and are to be followed up for an additional 16 weeks.

CT1812 is a ligand that targets the component 1 subunit of the sigma2/progesterone membrane receptor. It functions as a negative allosteric regulator, reducing the affinity of oligomeric Aβ and interfering with Aβ-induced synaptic toxicity [57]. START(COG0203) study (NCT05531656) is a phase 2, multicenter, randomized, double-blind, placebo-controlled trial that was initiated in September 2022 for evaluating the efficacy and safety of CT1812. START is comparing the effects of CT1812 (100 or 300 mg) with those of placebo over 18 months in 540 people with MCI or mild dementia due to AD. The SHINE (COG0201) study (NCT03507790) is a multicenter, randomized, double-blind, placebo-controlled, parallel-group, 36-week phase 2 study of two doses of CT1812 in adults with mild-to-moderate AD. The study is evaluating the safety, tolerability, pharmacokinetics, and efficacy of CT1812.

### Anti-tau therapy

Table 4 summarizes the ongoing phase 2 trials of anti-tau therapy.

**Table 4:** Ongoing phase 2 anti-tau therapy trials

Agent	Company	Synonyms	Therapeutic purpose	ClinicalTrials.gov identifier	Status
ACI-35	AC Immune SA	Tau Morphomer	Immunotherapy (passive)	NCT04445831	Active, not recruiting
Bepranemab	Hoffmann-La Roche, UCB S.A	UCB0107	Immunotherapy (passive)	NCT04867616	Active, not recruiting
BIIB080(CELIA)	Biogen, IONIS Pharmaceuticals	IONIS-MAPTRx, ISIS 814907	Tau DNA/RNA-based	NCT05399888	Recruiting
E2814 (DIAN-TU-001 (E2814))	Eisai		Immunotherapy (passive)	NCT05269394	Recruiting
JNJ-63733657 (AUTONOMY)	Janssen		Immunotherapy (passive)	NCT04619420	Recruiting
LY3372689	Eli Lilly & Co		Tau small molecule	NCT05063539	Active, not recruiting

Bepranemab (UCB0107) is a monoclonal IgG4 antibody that targets a central tau epitope. An ongoing phase 2 trial (NCT04867616) enrolling 421 participants with prodromal or mild AD is investigating the safety, tolerability, and efficacy of bepranemab. After an 80-week double-blinded treatment period, the participants are eligible to enter a 48-week open-label extension period, in which they are to receive bepranemab treatment for 44 weeks. Subsequently, they are to participate in a safety evaluation visit 20 weeks after the last infusion. The primary outcome measure is the CDR-SB score.

JNJ-63733657 is a humanized IgG1 monoclonal antibody that targets the microtubule-binding region of tau and prevents the cell-to-cell propagation of pathogenic tau aggregates. The AUTONOMY trial (NCT04619420) is an ongoing phase 2, randomized, double-blind, placebo-controlled, parallel-group multicenter study. Participants with early AD symptoms and a positive tau PET scan are randomized to groups receiving JNJ-63733657 or placebo. This trial is enrolling 420 participants and is expected to be completed by November 2025. The primary outcome measure is clinical decline, as determined using the iADRS.

ACI-35 is a liposome-based vaccine that targets pathological conformations of phosphorylated tau. A phase 1b/2a multicenter, double-blind, randomized, placebo-controlled trial (NCT04445831) was conducted to evaluate the safety, tolerability, and immunogenicity of various doses, regimens, and combinations of tau-targeting vaccines in individuals with early AD. The vaccines tested were JACI-35.054 and ACI-35.030 at various dose levels. The findings were presented at the 2022 CTAD conference. The results indicated that participants who received ACI-35.030 exhibited a strong and sustained immune response against pathological tau proteins (pTau) and nonphosphorylated tau (ePHF), particularly in the mid- and low-dose groups. Recipients of JACI-35.054 also displayed a robust immune response against ePHF and pTau, but without a clear dose–effect relationship. The trial has been conducted across nine centers in Finland, Sweden, the Netherlands, and the United Kingdom and is expected to be completed by October 2023.

E2814 is a monoclonal IgG1 antibody that targets an HVPGG epitope in the microtubule-binding domain of tau, prevents cell-to-cell propagation, and mediates the clearance of pathogenic tau proteins. The DIAN-TU-001 (E2814) trial (NCT05269394) is a phase 2/3 multicenter, randomized, double-blind, placebo-controlled platform trial of potential disease-modifying therapies with biomarker, cognitive, and clinical endpoints. The trial is enrolling patients with dominantly inherited AD. The study design involves the use of the anti-amyloid antibody lecanemab. Some participants are receiving a matching placebo plus lecanemab, whereas others are receiving concurrent therapy with E2814 plus lecanemab.

LY3372689 is a small-molecule inhibitor of O-GlcNAcase, which promotes tau glycosylation and prevents tau aggregation [58]. A phase 2 trial (NCT05063539) was initiated in September 2021 for assessing the safety, tolerability, and efficacy of LY3372689 in 330 patients with early symptomatic AD with progressive memory changes for ≥ 6 months and who met the criterion of having a positive flortaucipir-PET scan.

BIIB080 is a tau DNA/RNA-based antisense oligonucleotide that inhibits the translation of tau mRNA into protein, thus suppressing tau expression. CELIA (NCT05399888) is an ongoing phase 2 trial that is aiming to determine whether BIIB080 can delay AD progression in comparison with placebo and to identify the most effective dose of BIIB080. In March 2019, Biogen/Ionis performed a 4-year open-label extension trial of quarterly injections for individuals who completed the randomized portion of the trial. The initial data of this trial were reported at the Alzheimer’s Association International Conference (2021), revealing no serious adverse events from the intrathecal injection of BIIB080 at either of three doses every month for 3 months or two high-dose injections 3 months apart. BIIB080 led to a dose-dependent reductions of 30%–50% in total tau and pTau181 levels in CSF.

### Neuroprotectors and cognitive enhancers

Table 5 summarizes the ongoing phase 3 trials for therapies other than anti-amyloid/tau treatment.

**Table 5:** Ongoing phase 3 trials for therapies other than anti-amyloid/tau treatment

Agent	Mechanism of action	Target type and therapeutic purpose	ClinicalTrials.gov identifier	Status
ATH-1017 (fosgonimeton)	Enhance the activity of hepatocyte growth factor and its receptor	Neuroprotective; disease-modifying therapy	NCT04488419	Recruiting
AR1001	Inhibit phosphodiesterase 5 protein	Cognitive enhancer	NCT05531526	Recruiting
BPDO-1603	Undisclosed	Cognitive enhancer	NCT04229927	Unknown
Buntanetap	Suppress the translation of the mRNAs of neurotoxic aggregating proteins	Neuroprotective	NCT05686044 (Phase 2/3)	Not yet recruiting
Caffeine	Antagonist of adenosine receptors	Cognitive enhancer	NCT04570085	Recruiting
Hydralazine hydrochloride	(1) Activate the Nrf2 pathway, (2) rejuvenate mitochondria, (3) activate autophagy	Neuroprotective	NCT04842552	Recruiting
KarXT(xanomeline-trospium)	Muscarinic receptor agonist	Cognitive enhancer	NCT05511363	Recruiting
Masitinib	Tyrosine kinase inhibitor	Anti-inflammatory	NCT05564169	Recruiting
Masupirdine	Selective 5‐HT6 receptor antagonist	Neuropsychiatric symptom (agitation)	NCT05397639	Recruiting
Metformin	Antidiabetic	Cognitive enhancer	NCT04098666 (Phase 2/3)	Recruiting
Nabilone	Partial agonist of cannabinoid receptor 1 & 2	Neuropsychiatric symptom (agitation)	NCT04516057	Recruiting
NE3107	Blood–brain permeable anti-inflammatory insulin sensitizer that binds ERK	Anti-inflammatory	NCT04669028	Active, not recruiting
Nilotinib	Tyrosine kinase inhibitor	Neuroprotection	NCT05143528	Not yet recruiting
Piromelatine	Melatonin MT1 Feb Mar and serotonin 5-HT-1A JanD receptor agonist	Cognitive enhancer	NCT05267535 (Phase 2/3)	Recruiting
Semaglutide	Synthetic, long-acting analog of glucagon-like peptide-1	Neuroprotection	NCT04777396	Active, not recruiting
			NCT04777409	Active, not recruiting
Tricaprilin	Semisynthetic medium-chain triglyceride; alternative energy substrate for the brain	Cognitive enhancer	NCT04187547	Enrolling by invitation

*ERK* Extracellular signal-regulated kinase

The active metabolite of fosgonimeton (ATH-1017) is a positive modulator of hepatocyte growth factor (HGF)/MET signaling [59]. A phase 3 trial of fosgonimeton (NCT04488419) was initiated in September 2020 and is expected to be completed in February 2024. This study is evaluating the safety and efficacy of fosgonimeton in participants with mild-to-moderate AD, with double-blind, parallel-arm treatment implemented for 26 weeks. The primary outcome measure is the overall treatment effect of fosgonimeton, as measured using the Global Statistical Test, which combines cognition (ADAS-Cog) and function (ADCS-ADL) scores.

AR-1001 selectively inhibits phosphodiesterase 5 and suppresses cGMP hydrolysis, resulting in the activation of protein kinase G and the increased phosphorylation of the cAMP-responsive element-binding protein at Ser133. It can rescue long-term potentiation impairment and cognitive dysfunction in animal models of AD [60]. A phase 3 trial of AR-1001 (NCT05531526) was started in December 2022 and is estimated to be completed in December 2027. The study aims to evaluate the efficacy and safety of AR1001 in participants with early AD. The primary outcome measure is the change in the CDR-SB from baseline to week 52.

BPDO-1603 is a potential cognitive-enhancing drug for AD, but its mechanism of action remains unknown [61]. A phase 3 trial of BPDO-1603 (NCT04229927) was started in February 2020 and is estimated to be completed in March 2023. The study has been undertaken to evaluate the efficacy and safety of BPDO-1603 in patients with moderate-to-severe AD. The primary outcome measures are the change in Severe Impairment Battery total scores from baseline to week 24, and CIBIC-plus total scores at week 24.

Buntanetap is a novel translational inhibitor of multiple neurotoxic proteins, including APP, tau, and α-synuclein, by enhancing the binding of the atypical iron response element in the 5′UTR regions of the mRNA of the neurotoxic proteins to iron regulatory protein 1 [62]. In February 2023, phase 2 and 3 trials (NCT05686044) were initiated to measure the efficacy and safety of three doses of buntanetap in comparison with placebo in participants with mild-to-moderate AD. The primary outcome measures are ADAS-Cog and ADCS Clinical Global Impression of Change (ADCS-CGIC) scores.

Caffeine is an adenosine receptor antagonist that has been reported to be associated with slower cognitive decline and lower cerebral amyloid accumulation [63]. A phase 3 trial of caffeine (NCT04570085) was started in March 2021 to evaluate the efficacy of 30 weeks of caffeine intake in comparison with placebo on cognitive decline in patients with mild-to-moderate AD dementia (Mini-Mental State Examination scores: 16–24). The primary outcome measure is changes in neuropsychological test battery scores between the randomized value and the value after 30 weeks of treatment.

Hydralazine may have anti-neurodegenerative effects because it activates the Nrf2 pathway, which involves more than 200 antioxidant proteins; improves mitochondrial function; and increases respiration capacity and the production of adenosine triphosphate; hydralazine also activates autophagy, which aids in the clearance of intracellular aggregates [64–66]. A phase 3 trial of hydralazine (NCT04842552) was started in August 2021 and is anticipated to be completed in December 2023. The study is comparing the effects of 75 mg hydralazine versus placebo in patients with mild-to-moderate AD. Various cognitive and function tests, including olfactory tests, biochemical analyses, and adverse effect monitoring, are being conducted regularly during follow-up.

KarXT (xanomeline-trospium), comprised of muscarinic agonist xanomeline and muscarinic antagonist trospium, is designed to preferentially activate muscarinic receptor in the CNS and ameliorate the peripheral muscarinic side effects. It is reported that KarXT improves cognition in patients with AD and schizophrenia [67]. A 38-week phase 3 trial comparing the effects of KarXT (NCT05511363) and placebo in participants with psychosis associated with AD dementia was started in August 2022. The trial is analyzing the time from randomization to relapse (primary outcome) as well as the time from randomization to discontinuation for any reason and the safety and tolerability of KarXT (secondary outcomes).

Metformin, a commonly prescribed antidiabetic medication, has been reported to improve cognition or mood in many neurological disorders [68, 69]. A phase 3 trial of metformin (NCT04098666) was started in March 2021 and is anticipated to be completed in April 2026. The primary outcome measure is the total recall of the Free and Cued Selective Reminding Test at 24 months.

Nilotinib is a tyrosine kinase inhibitor that preferentially targets discoidin domain receptors and can effectively reduce the occurrence of misfolded proteins in animal models of neurodegeneration by crossing the blood–brain barrier and promoting Aβ and tau degradation [70]. A phase 3 trial (NCT05143528) was initiated in February 2022 to investigate the safety and efficacy of nilotinib BE (bioequivalent) in individuals with early AD. The primary outcome measure is changes in CDR-SB scores between baseline and week 72.

Piromelatine is a melatonin MT1/2/3 and serotonin 5-HT-1A/1D receptor agonist and was developed as a treatment for mild AD [71]. In May 2022, a randomized trial (NCT05267535) was initiated in 225 noncarriers of a specific polymorphism, and these participants with mild dementia due to AD are allocated at a ratio of 1:1 to receive piromelatine or placebo for 26 weeks. A 12-month extension involves treating the placebo group with piromelatine to assess the drug’s disease-modifying effects. The primary analysis will be conducted after the initial 26 weeks. If efficacy is not confirmed, the study is to end without the extension phase.

Semaglutide is a peptidic GLP-1 receptor agonist that may regulate the aggregation of Aβ in AD. GLP-1 receptors are involved in cognition, synaptic transmission in hippocampal neurons, and cell apoptosis; thus, they may serve as targets for exploring candidate drugs with neuroprotective and cognition-enhancing effects [72]. A phase 3 trial of semaglutide (NCT04777396) was started in May 2021 to investigate the efficacy of semaglutide in individuals with early AD. The primary outcome measure is changes in the CDR-SB score from baseline to week 104.

Tricaprilin, a semisynthetic medium-chain triglyceride, is hydrolyzed to octanoic acid after administration and is further metabolized to ketones, which serve as an alternative energy substrate for the brain [73]. Therefore, tricaprilin can be used as a ketogenic source for the management of mild-to-moderate AD. A phase 3 trial (NCT04187547) was started in June 2022 to evaluate the efficacy and safety of tricaprilin in participants with mild-to-moderate AD. The primary outcome measure is changes in ADAS-Cog scores from baseline to week 20.

### Anti-neuroinflammation therapy

Masitinib, an oral tyrosine kinase inhibitor, exerts effects by inhibiting mast cell and microglia/macrophage activity, with significant CNS penetration [74]. It is currently undergoing a phase 3 trial (NCT05564169) with 600 participants, employing a randomized, double-blind, placebo-controlled, parallel-group design over 24 weeks, followed by a 24-week extension phase. Quadruple masking ensures blinding. The study aims to evaluate Masitinib as an adjunct therapy for mild to moderate AD. Estimated to conclude on December 15, 2025, the trial assesses primary outcomes through changes from baseline in ADAS-Cog-11 and ADCS-ADL scores, measuring cognitive and functional abilities, respectively.

NE3107 is an anti-inflammatory insulin sensitizer that can cross the blood–brain barrier and bind to ERK. NE3107 can selectively inhibit inflammation-driven ERK- and NF-κB-stimulated inflammatory mediators, including TNF-α, without disturbing their homeostatic functions [75]. A multicenter phase 3 trial (NCT04669028) was started in August 2021 to investigate the safety and efficacy of NE3107 at 20 mg that was orally administered twice daily versus placebo in adult participants with mild-to-moderate AD. The primary outcome measures are changes in ADAS-Cog12 and ADCS-CGIC scores from baseline to week 30 [76].

### BPSD-relieving therapy

Masupirdine, a selective 5‐HT6 receptor antagonist with favorable physicochemical properties and absorption, distribution, metabolism, and excretion properties, may have beneficial effects on agitation, aggression, and psychosis in patients with moderate AD [77]. A phase 3 trial (NCT05397639) was started in November 2022 to evaluate the efficacy, safety, tolerability, and pharmacokinetics of masupirdine in comparison with placebo for treating agitation in participants with AD dementia. The primary outcome measure is the change in the score of the Cohen–Mansfield Agitation Inventory from baseline to week 12.

Nabilone is a partial agonist of cannabinoid receptor 1 (CB1) and CB2 in the brain and in peripheral tissues, and it has been reported to provide effective treatment for agitation in patients with AD [78]. A phase 3 trial (NCT04516057) was started in February 2021 to investigate whether nabilone is an effective treatment for agitation in AD patients. The primary outcome measure is agitation (Cohen–Mansfield Agitation Inventory) between baseline and week 8.

### Phase 4 and repurposing trials

Table 6 summarizes ongoing phase 4 trials.

**Table 6:** Ongoing phase 4 trials

Agent	Mechanism of action	Target type and therapeutic purpose	ClinicalTrials.gov identifier	Status
Escitalopram Oxalate	Selective-serotonin reuptake inhibitor	Neuropsychiatric symptoms (depression)	NCT05004987	Recruiting
Sodium Oligomannate Capsule (GV-971)	Neuroinflammation inhibitor, Aβ fibril formation inhibitor	Anti-neuroinflammation, amyloid-related	NCT05181475	ACTIVE, NOT RECRUITING
			NCT05430867	Not yet recruiting
Spironolactone	Aldosterone mineralocorticoid receptor antagonist	Anti-neuroinflammation	NCT04522739	Recruiting

Escitalopram, a selective-serotonin reuptake inhibitor, is a commonly used antidepressant. It ameliorates cognitive impairment and could selectively attenuate phosphorylated tau accumulation in stressed rats by regulating hypothalamic–pituitary–adrenal axis activity and the insulin receptor substrate/glycogen synthase kinase-3β signaling pathway [79]. A phase 4 trial (NCT05004987) was started in February 2022 to investigate whether a reduction in depressive symptoms owing to the administration of escitalopram oxalate is associated with the normalization of AD biomarkers in CSF and inflammatory markers in the peripheral blood. The primary outcome measures are changes in CSF Aβ40 and Aβ42 levels, vascular dysfunction biomarker levels, and scores of the Montgomery–Asberg Depression Ratio Scale at week 8.

Sodium oligomannate (GV-971), a marine-derived oligosaccharide, can reconstitute the gut microbiota, reduce bacterial metabolite–driven peripheral infiltration of immune cells into the brain, inhibit amyloid-β fibril formation, and inhibit neuroinflammation in the brain, as demonstrated in animal studies [80, 81]. A phase 4 trial (NCT05181475) was initiated in December 2021 to examine the long-term efficacy and safety of GV-971 as well as changes in blood and gut microbiota biomarkers and thereby validate its mechanism of action and establish guidance for the more rational use of drugs in clinical practice. The primary outcome measure is changes in ADAS-Cog11 scores from baseline to week 48. Another phase 4 trial was started in July 2022 and is comparing the efficacy and safety of memantine and GV-971 monotherapy and combination therapy in patients with moderate-to-severe AD. The primary outcome measure is changes in cognitive function at weeks 12, 24, 36, and 48.

Spironolactone, an aldosterone mineralocorticoid receptor antagonist, has been commonly used to treat cardiovascular diseases, including hypertension. It has anti-inflammatory effects on the peripheral tissues and central nervous system and therefore may have beneficial effects on neurological disorders [82]. A phase 4 trial (NCT04522739) was started in September 2022 to investigate whether spironolactone can be tolerated by older Black American adults with MCI and to determine its effect on memory and thinking abilities, as measured by participant performance on cognitive tests. The primary outcome measures are the number of adverse events and the attrition rate.

### Published results

Among the clinical trials newly registered in the last 4 years, four articles pertaining to two trials have been published in peer-reviewed scientific journals. The characteristics of the published randomized controlled trials are summarized in Table 7 [43, 53, 83, 84]. Two articles reported the results of NCT03887455 [43, 84], and the other two reported the results of NCT01760005 [53, 83]. The articles were published between 2018 and 2023. The results of both NCT03887455 (Clarity AD) and NCT01760005 have been discussed in the anti-amyloid section. The methodological quality of these studies is summarized in Table 8. Both trials (NCT03887455 and NCT01760005) had a overall low risk of bias [43, 53, 83, 84].

**Table 7:** Characteristics of published randomized controlled trials

Author [Year]; Country	Selection criteria	Number of patients (% male)	Age, year, mean ± SD	Intervention	Duration	Follow up period	Outcome
Dyck et al. [2023]; United States of America	RCT; patients 50 to 90 years of age with early AD (MCI or mild dementia due to AD)	L: 859 (48.4) P: 875 (47.0)	L: 71.4 ± 7.9 P: 71.0 ± 7.8	L: 10 mg of intravenous lecanemab per kilogram every 2 weeks P: Placebo	18 months (79 weeks)	91 weeks	(1) CDR-SB Score (difference: − 0.45; 95% CI: -0.67 to − 0.23) (2) Amyloid Burden on PET (difference: − 59.1; 95% CI: -62.6 to − 55.6) (3) ADAS-Cog14 Score (MD: − 1.44; 95% CI: − 2.27 to -0.61) (4) ADCOMS (MD: -0.050; 95% CI: − 0.074 to − 0.027) (5) ADCS-MCI-ADL Score (MD: 2.0; 95% CI: 1.2 to 2.8) (6) adverse events (L: infusion-related reactions in 26.4% of participants and amyloid-related imaging abnormalities with edema or effusions in 12.6%.)
Tahami Monfared et al. [2023]; United States of America	RCT; the same group of patients from Dyck et al	L: 859 (48.4) P: 875 (47.0)	L: 71.4 ± 7.9 P: 71.0 ± 7.8	L: 10 mg of intravenous lecanemab per kilogram every 2 weeks P: Placebo	18 months (79 weeks)	91 weeks	(1) quality-adjusted life-years [L: 4.39 (4.27–4.50); P: 3.68 (3.60–3.77)] (2) mean time to progression to AD dementia [L: 8.64 (7.91–9.57); P: 5.69 (5.32–6.16)] (3) years in institutional care [L: 0.78 (0.69–0.87); P: 0.89 (0.80–0.98)] (4) health outcomes
Salloway et al. [2021]; Australia, Canada, France, Spain, United Kingdom, and the United States of America	RCT; participants known to have or at-risk for a DIAD mutation-60% asymptomatic	AG: 52 (60) AS: 50 (42) P: 40 (45)	AG: 46.0 ± 10.8 AS: 42.5 ± 9.5 P: 44.2 ± 9.6	AG: 225 mg of gantenerumab (subcutaneously, every 4 weeks) = > 1,200 mg from year 4 AS: 400 mg of solanezumab (subcutaneously, every 4 weeks) = > 1,600 mg from year 5	4–7 years	NA	(1) Digit Symbol scores (2) MMSE scores; (3) Logical Memory scores; (4) ISLT Delayed Recall scores; (5) CDR-SB scores; (6) FAS scores; (7) brain amyloid burden; (8) CSF total Aβ42; (9) CSF phospho-tau181; (10) CSF total tau; (11) CSF NfL
Bateman et al. [2018]; Australia, Canada, France, Spain, United Kingdom, and the United States of America	RCT; the same group of patients from Salloway et al	AG: 52 (60) AS: 50 (42) P: 40 (45)	AG: 46.0 ± 10.8 AS: 42.5 ± 9.5 P: 44.2 ± 9.6	AG: 225 mg of gantenerumab (subcutaneously, every 4 weeks) = > 1,200 mg from year 4 AS: 400 mg of solanezumab (subcutaneously, every 4 weeks) = > 1,600 mg from year 5	4–7 years	NA	(1) Cognitive composite performance; (2) Proportional hypothetical treatment effects

*L* lecanemab group; *P* placebo group

DIAN-TU-001 mutation carriers: *AG* active gantenerumab group, *AS* active solanezumab group, *P* shared placebo group

*CDR-SB score* Clinical Dementia Rating – Sum of Boxes, *ADAS-Cog14* Alzheimer’s Disease Assessment Scale-Cognitive subscale 14, *ADCOMS* AD Composite Score;

*ADCS-MCI-ADL Score* Alzheimer's Disease Cooperative Study – Activities of Daily Living Scale for use in Mild Cognitive Impairment, *MMSE* Mini-Mental State Examination;

*ISLT* International Shopping List Test, *FAS* Functional Assessment Scale; CSF: cerebrospinal fluid, *NfL* Neurofilament light polypeptide

**Table 8:** Methodological quality assessment of published randomized controlled trials using the Cochrane risk of bias tool (2^nd^ version)

Study	Bias from the randomization process	Bias caused by deviations from intended interventions	Bias caused by missing outcome data	Bias in measurement of the outcome	Bias in selection of the reported results	Overall risk of bias
Dyek [2023]	Low risk	Low risk	Low risk	Low risk	Low risk	Low risk
Tahami Monfared 2023]	Low risk	Low risk	Low risk	Low risk	Low risk	Low risk
Salloway [2021]	Low risk	Low risk	Low risk	Low risk	Low risk	Low risk
Bateman [2018]	Low risk	Low risk	Low risk	Low risk	Low risk	Low risk

## Discussion

Our understanding of AD originated from clinical research, and how pathological findings are associated with clinical presentation of AD has continued to intrigue the neuroscience research community over the past century. DMTs have become the core of new drug development, and the accumulation of knowledge is leading to the evolution of diagnostic criteria and clinical outcome measurements. The view of clinical outcomes has shifted from considering them as solely determinative to considering them to be just one of the determinants. In accordance with the 2018 NIA-AA Research Framework criteria [25] or the new 2023 NIA-AA revised criteria for AD [26], the incorporation of biomarkers is necessary in clinical practice.

This review documented that in terms of the number of AD drug trials and the number of recruited participants, the majority of trials continue to focus on mechanisms involving amyloid and tau. Our 2020 report highlighted that due to the failure of early anti-amyloid trials to achieve their intended outcomes, particularly studies involving BACE inhibitors and monoclonal antibodies, some have questioned whether amyloid remains clinically relevant in AD. This shift in perspective has led to a change in the focus of research toward populations in the prodromal or preclinical stage with positive results for diagnostic biomarkers. Additionally, the validity of the amyloid hypothesis has been contested, resulting in a significant reduction in the number of anti-amyloid phase 3 trials since 2019. However, the targets of both phase 1 and phase 2 trials are diverse, with a noticeable increase in the number of phase 1 trials focusing on neuroprotection and phase 2 trials focusing on anti-neuroinflammation [85]. Since the positive outcomes in terms of slow decline in cognitive abilities in the lecanemab Clarity AD trial [43] and the donanemab trial TRAILBLAZER-ALZ [86], the impact of amyloid and consequent pathological alterations is likely to become the main focus of clinical trials. The incorporation of amyloid-related therapy either as an add-on or as a link to specific aspects of AD pathophysiology might become an important trend in clinical trials of new drugs in the future. However, despite this expansion of research areas, the scope of indications for novel anti-amyloid monoclonal antibody therapy remains limited. The mode of treatment administration and the high monitoring costs along with the need for specialized facilities and imaging scans remain challenges. Other unmet needs, such as addressing BPSD and enhancing cognitive function, necessitate pharmaceutical research. Examining drugs with diverse mechanisms necessitates thorough evaluation that extends beyond mere clinical measurements to encompass their intermediate impact on biomarkers. It is essential to investigate the potential synergy between a new drug and existing medications approved by the US FDA. This approach could even be extended to situations where adjuvant treatment, such as tau-related treatments, is provided after amyloid clearance has been achieved. Clinical trials related to AD have also exhibited a shift in focus toward the earlier stages of AD, such as MCI, or even cognitively healthy participants for developing prevention interventions.

## Conclusion

**Fig 2.**
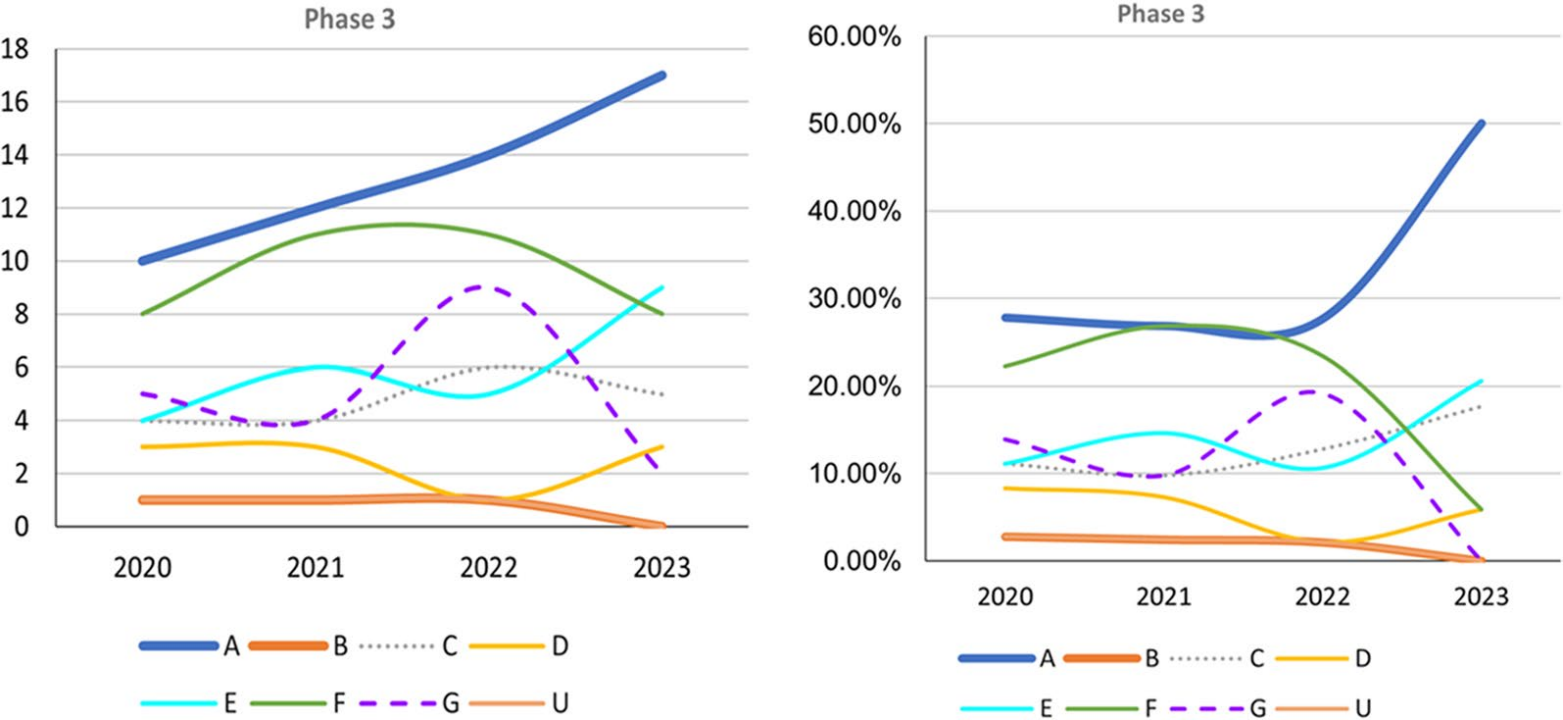
Trends in Phase 3 trials, 2020–2023, categorized according to event-related themes in ClinicalTrials.gov. Left: Number of Phase 3 trials. Right: Percentage of Phase 3 trials. *A* anti-amyloid therapy, *B* anti-tau therapy, *C* neuroprotection, *D* anti-neuroinflammation, *E* cognitive enhancer, *F* relief of behavioral psychological symptoms of dementia, *G* others, *U* undisclosed

**Fig. 3:**
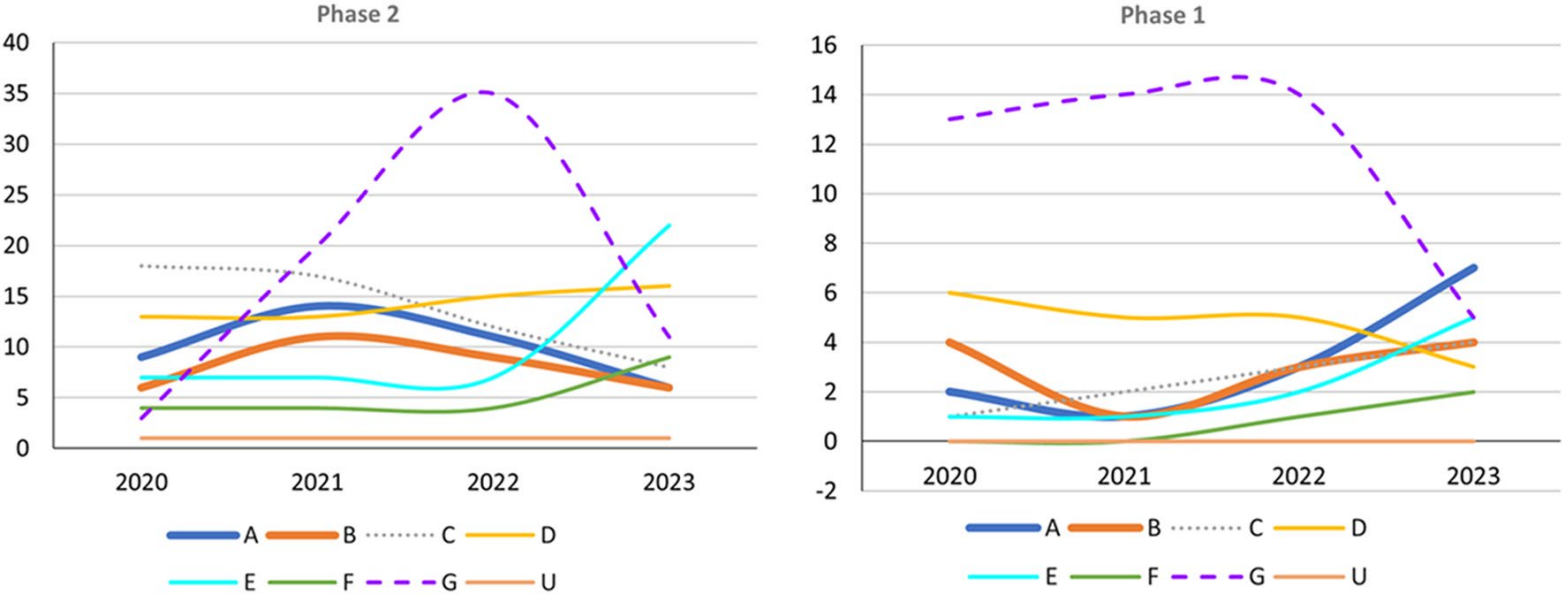
Trends in Phase 1 and 2 trials, 2020–2023, categorized according to event-related themes in ClinicalTrials.gov. Left: Number of Phase 2 trials. Right: Number of Phase 1 trials; *A* anti-amyloid therapy, *B* anti-tau therapy, *C* neuroprotection, *D* anti-neuroinflammation, *E* cognitive enhancer, *F* relief of behavioral psychological symptoms of dementia, *G* others, *U* undisclosed

Successful phase 3 trials such as Clarity AD (lecanemab) and EMERGE (aducanumab) have evaluated anti-amyloid treatment in mild AD (Fig. 2). Trials that do not target specific pathophysiologies are becoming fewer in all phases (Figs. 2 and 3). However, an increasing number of early-phase trials of therapies for symptoms, including cognitive enhancers and agents for relieving BPSD, are being conducted. This reflects the unmet clinical need for such therapies (Figs. 2 and 3). Similarly, an increasing number of phase 1 trials involving DMTs, particularly those targeting both anti-amyloid and anti-tau mechanisms, has been noted, indicating the importance of basic research (Fig. 3). Outcome measurement tools have also become more diverse, which has enabled meaningful improvements in AD and the efficacy of treatments to be clearly determined in clinical trials. Overall, the field of AD clinical trials is evolving, and additional promising treatments for AD are likely to be developed in the near future.

## Data Availability

Not applicable.

## Abbreviations

Aβ: Amyloid-beta
Ach: Acetylcholine
AChE-Is: Cholinesterase inhibitors
AD: Alzheimer disease
ADAS-Cog 13: Alzheimer’s Disease Assessment Scale–Cognitive Subscale
ADAS-ADL-MCI: Alzheimer’s Disease Cooperative Study–Activities of Daily Living Inventory–Mild Cognitive Impairment Version
APOE: Apolipoprotein gene
APP: Amyloid precursor protein
ARIAs: Amyloid-related imaging abnormalities
ATN biomarkers: Amyloid, tau, and neurodegeneration biomarkers
AUR: Appropriate use recommendations
AV: Autophagic vacuoles
BACE1: Beta-secretase 1
BPSD: Behavioral psychological symptoms of dementia
CDR: Clinical Dementia Rating scale
CDR-SB: Clinical Dementia Rating scale Sum of Box
CGIC: Caregiver Global Impression of Change
CSF: Cerebrospinal fluid
CTAD: Clinical Trial of AD
DMTs: Disease-modifyung therapies
iADRS: Integrated Alzheimer’s Disease Rating Scale
IgG1: Immunoglobulin gamma 1
MCI: Mild cognitive impairment
MRI: Magnetic resonance imaging
NF-κB: Nuclear factor κB
NFTs: Neurofibrillary tangles
NPI-10: Neuropsychiatric Inventory
PET: Positron emission tomography
PSEN1: Presenilin-1
PSEN2: Presenilin-2
